# Diverging metabolic programmes and behaviours during states of starvation, protein malnutrition, and cachexia

**DOI:** 10.1002/jcsm.12630

**Published:** 2020-09-28

**Authors:** Brennan Olson, Daniel L. Marks, Aaron J. Grossberg

**Affiliations:** ^1^ Medical Scientist Training Program Oregon Health & Science University Portland OR USA; ^2^ Papé Family Pediatric Research Institute Oregon Health & Science University Portland OR USA; ^3^ Brenden‐Colson Center for Pancreatic Care Oregon Health & Science University Portland OR USA; ^4^ Department of Radiation Medicine Oregon Health & Science University Portland OR USA; ^5^ Cancer Early Detection Advanced Research Center Oregon Health & Science University Portland OR USA

**Keywords:** Starvation, Cachexia, Metabolism, Evolution, Protein malnutrition

## Abstract

**Background:**

Our evolutionary history is defined, in part, by our ability to survive times of nutrient scarcity. The outcomes of the metabolic and behavioural adaptations during starvation are highly efficient macronutrient allocation, minimization of energy expenditure, and maximized odds of finding food. However, in different contexts, caloric deprivation is met with vastly different physiologic and behavioural responses, which challenge the primacy of energy homeostasis.

**Methods:**

We conducted a literature review of scientific studies in humans, laboratory animals, and non‐laboratory animals that evaluated the physiologic, metabolic, and behavioural responses to fasting, starvation, protein‐deficient or essential amino acid‐deficient diets, and cachexia. Studies that investigated the changes in ingestive behaviour, locomotor activity, resting metabolic rate, and tissue catabolism were selected as the focus of discussion.

**Results:**

Whereas starvation responses prioritize energy balance, both protein malnutrition and cachexia present existential threats that induce unique adaptive programmes, which can exacerbate the caloric insufficiency of undernutrition. We compare and contrast the behavioural and metabolic responses and elucidate the mechanistic pathways that drive state‐dependent alterations in energy seeking and partitioning.

**Conclusions:**

The evolution of energetically inefficient metabolic and behavioural responses to protein malnutrition and cachexia reveal a hierarchy of metabolic priorities governed by discrete regulatory networks.

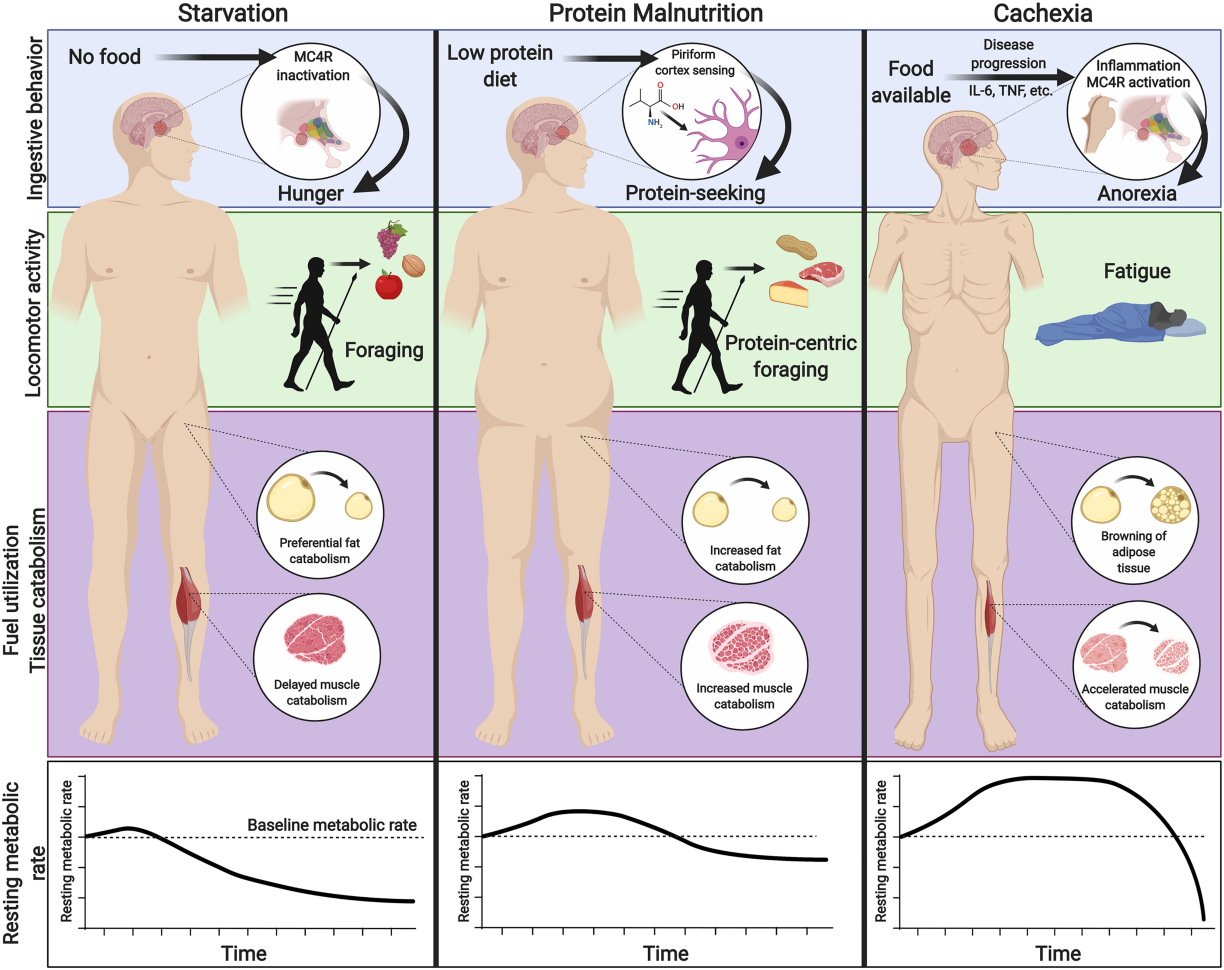

[Correction added on 28 October 2020 after first online publication: Summary figure has been added in this current version.]

## Introduction

Appropriate nutrient consumption is an essential process in sustaining normal biological processes. Both small and extreme fluctuations in caloric intake result in changes in metabolism and behaviour in effort to maintain organismal homeostasis. During calorically plentiful states, organisms activate energy‐consuming anabolic pathways and satiety behaviours, while states of caloric deprivation result in energy‐liberating catabolic pathways and foraging behaviours.^1^, ^2^ These metabolic programmes and behaviours are evolutionarily conserved responses that were shaped to endure periods of famine.^3^ Specifically, starvation results in metabolic adaptations that decrease energy expenditure and conserve protein stores in order to preserve organ function, while increasing appetite and foraging behaviours in attempt to correct the underlying nutritional deficiency. However, in the context of protein‐specific malnutrition or disease‐associated cachexia, the metabolic and behavioural responses to nutrient insufficiency differ from simple starvation. For example, in both protein malnutrition and cachexia, catabolic processes are activated, and macronutrient intake is paradoxically suppressed, violating the rule of energy conservation. While decades of research demonstrate that the physiologic and behavioural responses activated during starvation serve to spare energy stores and restrict energy expenditure, recent discoveries regarding the physiology and behavioural neuroscience of protein malnutrition and cachexia reveal additional levels of metabolic regulation that lend insight into the pressures that guided metabolic pathway evolution.

**Figure 1:**
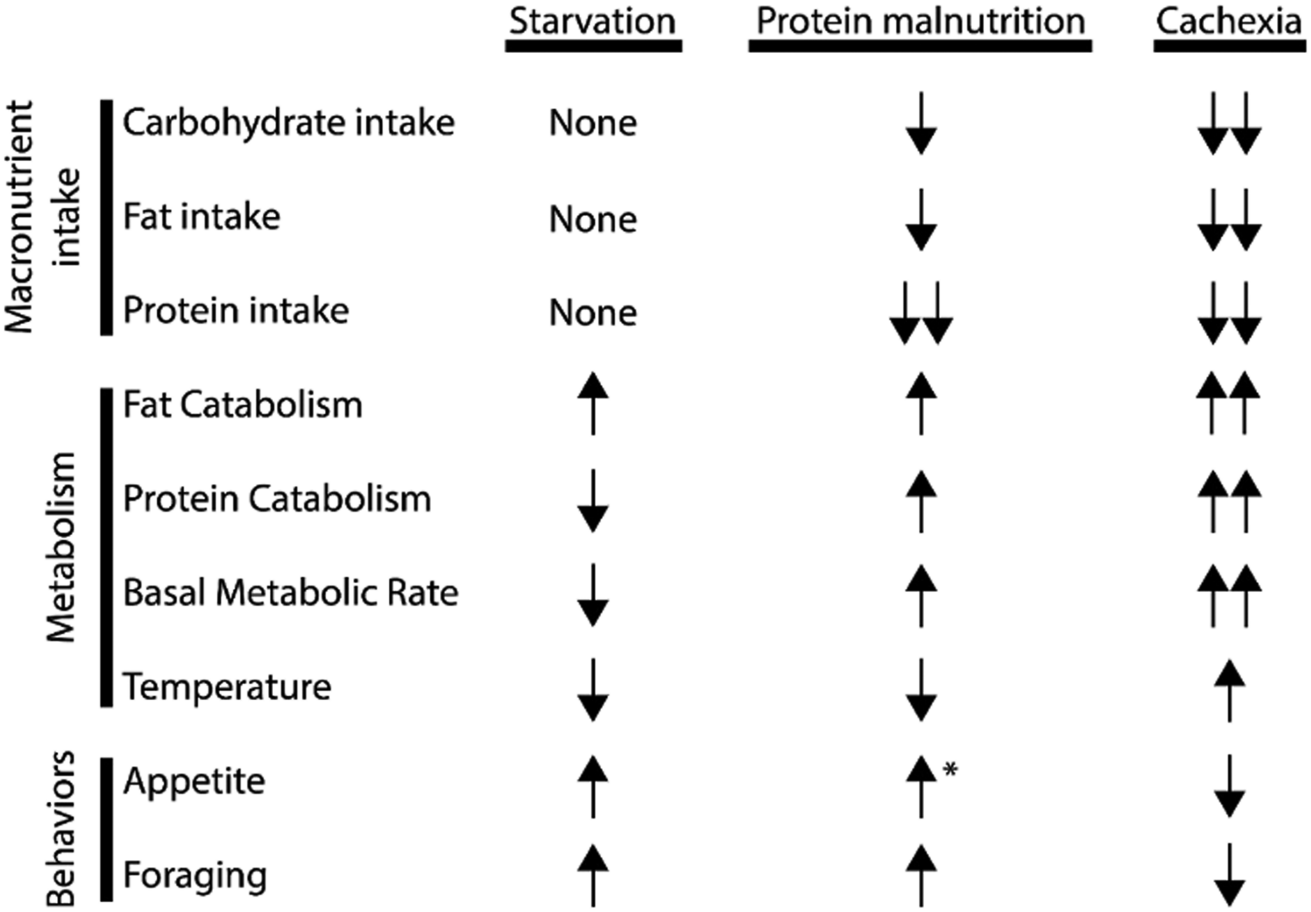
An overview of macronutrient intake, tissue metabolism, and behavioural changes observed during simple starvation, protein deficiency, and cachexia. *Increased appetite to protein‐rich foods, yet active rejection of protein‐poor foods.

Simple starvation, defined herein as pure caloric deficit in an otherwise healthy organism, activates programmes that prioritize metabolic efficiency, thereby promoting resilience and survival. Less is known about protein malnutrition and cachexia, and recent evidence suggests that the metabolic programmes of these states in response to caloric deficit are broadly inefficient. Furthermore, these three states of nutrient deprivation result in unique behavioural responses that either complement or contradict the overall nutrient requirement of the organism (*Figure* 1). In this review, we will discuss the disparate physiologic responses of the metabolic states of simple starvation, protein malnutrition, and cachexia, with a particular focus on our current understanding of metabolism, neurophysiology, and behavioural outputs.

## Starvation

Metabolic homeostasis is maintained by a well‐described network involving regions in the hypothalamus and brainstem that respond to hormonal and metabolic signals of both short‐term and long‐term energy supply and engage appropriate behavioural and physiologic programmes.^4^ Under physiologic conditions, mammals are able to match cumulative energy intake with energy expenditure with exceptional precision because of tight control of tissue metabolism and feeding behaviours by the central nervous system.^5^, ^6^ Because nutrient scarcity represented an existential threat to organisms throughout their evolution, these systems prioritize efficiency and energy storage to maximize both countermeasures against and resilience to undernutrition. This response is defined behaviourally by an increase in appetite and foraging behaviour and metabolically by a decrease in basal metabolism and preferential catabolism of adipose over lean tissue.^7^, ^8^ Although this imbalanced response to nutrient availability likely made humans more vulnerable to obesity in the context of high nutrient availability, it ensures that the long‐term effects of nutrient insufficiency are minimized. In this review, we use simple starvation to introduce the homeostatic response to nutritional insufficiency, which will then serve as our comparator when discussing the respective situations of protein malnutrition and cachexia.

### Ingestive behaviour

Secreted peripheral factors alter neuronal activity and feeding behaviours during starvation, with decades of research demonstrating the influence of gut‐secreted and fat‐secreted neuropeptides on the mediobasal hypothalamus (MBH).^9^ Here, we will briefly discuss the well‐studied endocrine molecules ghrelin and leptin that are known to play roles in driving behaviours of feeding at least in part through their direct, yet opposing, mechanisms on hypothalamic neurons. Other peripherally secreted hormones that influence food intake under physiologic conditions are summarized in *Table* 1. During fasting and starvation, the stomach releases the peptide hormone ghrelin, the only known circulating hormone that stimulates appetite. After secretion by the stomach, acylation of ghrelin is required for its binding to its receptor, the growth‐hormone‐secretagogue receptor, and for its ability to cross the blood–brain barrier.^10^ Once in the brain, ghrelin stimulates appetite through interaction with appetite‐regulating neurons in the MBH. These include neurons that inhibit food intake [pro‐opiomelanocortin (POMC) neurons], and appetite‐stimulating neurons expressing neuropeptide Y and agouti‐related protein (AgRP), known collectively as the melanocortin system. The melanocortin system exerts many of its effects through regulation of activity at the Type 4 melanocortin receptor (MC4R), which is expressed in numerous brain regions. POMC neurons release the MC4R agonist neurotransmitter alpha‐melanocyte stimulating hormone, whereas AgRP neurons directly inhibit POMC neuronal activity and also release the MC4R inverse agonist AgRP at most MC4R expressing neurons. Ghrelin induces food intake primarily via activation of neurons expressing neuropeptide Y/AgRP neurons in the arcuate nucleus of the MBH,^11^, ^12^, ^13^ which in turn send projections to numerous nuclei within the hypothalamus, including the paraventricular, ventromedial, dorsomedial, and lateral hypothalamus, as well as nuclei outside of the hypothalamus, including the nucleus of *tractus solitarii* and parabrachial nucleus (PBN).^14^ Conversely, the anorexigenic adipokine leptin is markedly reduced during starvation.^15^ Leptin functions as a long‐term signal of energy status, with circulating levels proportional to total adipose stores.^16^ Whereas the presence of leptin is permissive of normal caloric intake and neuroendocrine function, a fall in leptin levels signals a loss of long‐term energy stores. Correspondingly, decreased leptin is associated with decreased anorexigenic POMC neuronal activity, thereby triggering hunger and accompanying physiologic responses during starvation.^17^ This neuroendocrine interplay between rising levels of ghrelin and falling levels of leptin synergistically increases appetite during starvation. This intricate balance between upregulation of orexigenic and downregulation of anorexigenic molecules is a unifying theme of starvation neurophysiology.

**Table 1:** Additional endocrine molecules that mediate food intake

Hormone	Source	Signalling mechanism(s)
Cholecystokinin (CCK)	Enteroendocrine cells of the duodenum and jejunum	Peripheral vagal afferent receptors and transmission of signals to nucleus of the solitary tract; Melanocortin system^198^
Glucagon‐like peptide‐1 (GLP1)	L cells of distal small and large intestine	nucleus of the solitary tract in the brainstem and the paraventricular nucleus of the hypothalamus; glucose regulation^199^, ^200^
Peptide YY (PYY)	endocrine L cells of the gut	Hypothalamic melanocortin system*; Aversive response; protein‐dependent satiety^201^, ^202^
Glucocorticoids	Adrenal gland	Unclear, but potentially permissive in the orexigenic effect of AgRP in the hypothalamus^203^, ^204^
Insulin	Endocrine pancreas	Hypothalamic melanocortin system^205^, ^206^

### Locomotor activity

The regulation of activity in response to starvation is somewhat more complex than that of appetite. On one hand, voluntary activity increases energy usage and exacerbates energy debt in the absence of food intake. Yet, for nearly all of human existence, survival depended on the ability to forage or to efficiently locate, acquire, and consume food.^18^ As such, foraging is deemed an obligate life history strategy, and a species' ability to recognize when foraging is beneficial or detrimental is a part of its evolutionary code.^19^ In general, starvation increases foraging behaviours when the likelihood of a meal is increased, yet limits foraging and movement when prey or food is limited.^20^, ^21^ During calorie deprivation, hyperactivity and increased foraging behaviour are readily observed in rodents, wherein they exhibit stereotypic food anticipatory activity in the hours preceding mealtime.^22^ Although the neural pathways underlying this response are incompletely understood, this behaviour is associated with concurrent increases in hypothalamic turnover of norepinephrine, dopamine, and serotonin.^23^ The neuropeptide orexin‐A, released by neurons located in the lateral and perifornical hypothalamus, is required for fasting‐associated activity increases. Furthermore, recent mouse work demonstrated that AgRP neurons in the MBH are themselves activated during starvation and are capable of driving foraging behaviour.^24^ Orexin neurons reciprocally regulate both hypothalamic AgRP neurons and catecholaminergic neurons in the locus coeruleus, establishing a brainstem‐to‐hypothalamus arousal loop that appears to mediate fasting‐associated foraging.^25^, ^26^ The degree of hyperactivity and foraging is balanced between fear of predation and likelihood of feeding, and these processes are influenced, in part, through amygdala circuitry.^27^

Rodents will increase their locomotor activity (foraging) when calorie availability is restricted, but access to at least some nutrition is maintained. In contrast, complete removal of food causes a triphasic response in weight loss and locomotor activity in rodents.^28^, ^29^, ^30^ The short‐lived first phase is defined by early rapid weight loss and a decline in daily activity within 24 h of fasting initiation. During a prolonged second phase, ongoing suppression of activity is associated with low rates of protein turnover, high dependence on lipid oxidation, and relatively steady body mass. Upon exhaustion of adipose depots, fasted rodents then show a profound rise in locomotor activity in the third phase, associated with rapid weight loss and protein catabolism.^31^ The duration of the energy‐conserving second phase is age dependent, longest in older animals that have larger adipose depots. Similar responses were shown in other species, most notably migratory birds and emperor penguins, in which the metabolic shift from lipid to protein catabolism is a signal of expiring energy stores that switches behavioural programme from conservation to active foraging.^32^, ^33^ Collectively, these observations demonstrate the clear link between voluntary activity and energy balance and reveal an evolutionarily conserved mechanism whereby activity is regulated both by food availability and by long‐term energy stores.

### Resting metabolic rate

Energy conservation is a key component of the adaptive response to starvation. Resting metabolic rate (RMR), the energy used to maintain body temperature, repair organs and tissues, maintain ion gradients, and support cardiorespiratory function, accounts for approximately two‐thirds of total energy expenditure.^34^ Therefore, the RMR represents the greatest potential reservoir for energy conservation. Indeed, decades of research demonstrate that one of the main adaptations in humans and other species to nutrient deprivation is to reduce RMR. RMR is proportional to an animal's lean body mass, as lean tissues are far more metabolically active than adipose tissue. The suppression of RMR seen in response to even prolonged starvation exceeds that which can be explained by pure loss of lean mass, indicating that RMR depression is an active conservation strategy. This is known as ‘adaptive thermogenesis’, because heat generation is the principal component of resting energy expenditure that is modulated in response to feeding. One of the earliest reports of this process showed the basal metabolic rate (equal to the RMR upon waking, while fasted and at rest) of a man who fasted for 42 days, with intake limited to water, lemonade, or beer. Basal metabolism progressively decreased until the end of his fast, at which time, his resting energy expenditure was approximately half of that in the fed state.^35^ Similarly, the Minnesota experiment challenged normal weight participants through stages of semi‐starvation, restricted refeeding, and *ad libitum* refeeding, demonstrating excess suppression of RMR during undernutrition.^36^ Rats and mice also reduce thermogenesis response to fasting, suggesting that this is an evolutionarily conserved mechanism to preserve body mass.^37^, ^38^ Suppression of thermogenesis can preserve mass over a large range of caloric deficits, perhaps most recognizably in the context of failure to lose weight during dieting.^39^

The RMR is largely under the control of the sympathetic nervous system (SNS). Catecholamines released into the synapse from noradrenergic nerve terminals or systemically from the adrenal medulla increase the rate of cellular metabolism and mobilize fuel stores by stimulating lipolysis in adipocytes and glycogenolysis and gluconeogenesis from the muscle and liver.^40^ The SNS is responsive to nutritional status—engaged by overfeeding and suppressed by fasting.^41^ This response is seen in the human studies cited above, wherein fasting decreased resting heart rate, a surrogate for decreased sympathetic tone, in addition to its effects on thermogenesis. Indeed, fasting mice and rats also exhibit decreases in heart rate and blood pressure, consistent with decreased SNS activity.^42^, ^43^ Fasted rats have lower levels and turnover of norepinephrine in the heart, liver, pancreas, and other sympathetically innervated tissues as compared with fed rats.^44^ Although these cardiovascular effects can themselves conserve energy, the SNS has direct effects on thermogenesis mediated primarily by brown and white adipose tissue (WAT) via the β3 adrenoceptor.^45^ SNS activation stimulates uncoupled oxidative respiration via the expression of uncoupling protein 1 (UCP1), leading to non‐shivering thermogenesis in brown adipose tissue (BAT).^46^ Simultaneously, adrenergic input induces lipolysis in WAT, thereby providing a fuel source for BAT thermogenesis.^47^ Although previously thought to only be found in infants, BAT has recently been identified as an important thermogenic tissue in adult humans, as well.^48^, ^49^, ^50^, ^51^, ^52^ Thus, the decreases in RMR induced by fasting appear to be largely mediated by decreased SNS activity and the resultant restriction of thermogenesis and cardiac output.

### Fuel utilization and tissue catabolism

**Figure 2:**
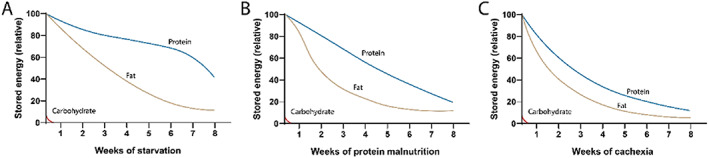
Relative rates of carbohydrate, fat, and protein catabolism during simple starvation (A), protein malnutrition (B), and cachexia (C).

The primary purpose of the metabolic response during fasting and starvation is to provide sufficient energy to the brain and other tissues critical for survival. During starvation, energy in the form of glucose is mobilized during early starvation, with ketone bodies serving as the primary energy source for the heart and brain in prolonged starvation.^53^ If fasting proceeds beyond one day in humans, or 8–12 h in mice, hepatic stores of glycogen are rapidly depleted, and catabolism of adipose and muscle tissue serve as the major sources of energy.^54^, ^55^ Because fat stores are limited in their ability to generate glucose, muscle catabolism is the primary source of hepatic and renal glucose production during starvation through liberation of gluconeogenic amino acids.^56^ However, proteins are not a substantial stored energy reserve, and humans evolved to preserve protein by shifting our fuel utilization from glucose to ketone bodies after just 2 days of starvation.^57^ These ketone bodies are produced by the liver from lipolysis‐liberated fatty acids and significantly curtail muscle catabolism during starvation.^58^ Indeed, humans preferentially catabolize fat stores over skeletal muscle mass during prolonged caloric deficit.^1^ If starvation persists after fat stores are depleted, protein catabolism accelerates and can lead to severe wasting, organ failure, and ultimately death.^59^, ^60^ The preferential catabolism of adipose tissue is largely driven by the endocrine response to starvation. In the short term, falling blood sugar is met with the counter‐regulatory endocrine response, including release of glucocorticoids, glucagon, and growth hormone, and a concomitant decrease in insulin.^61^, ^62^ This stimulates both gluconeogenesis and lipolysis, engaging a catabolic programme that redistributes stored energy in the absence of food intake.^63^ Although full discussion is outside of the scope of this review, a review of the major changes in circulating hormone levels is summarized in *Table* 2. Taken together, this strategic triaging of energy store utilization during starvation serves to reduce breakdown of proteins while providing adequate energy substrates for the brain and other tissues critical for survival (*Figure* 2A).

**Table 2:** Overview of peripheral hormone response during starvation, protein malnutrition, and cachexia

	Starvation	Protein malnutrition	Cachexia
Cortisol	*Increased* ^63^	*Increased* ^207^, ^208^	*Increased* ^96^, ^209^
Thyroid hormone	*Decreased* ^210^, ^211^, ^212^	*Decreased* ^213^	*Increased* ^a^ ^143^
Parathyroid hormone	*Increased* ^214^	*Increased* ^215^	Increased^138^, ^216^
Renin–angiotensin aldosterone	*Increased* ^217^, ^218^	*Increased; minimal excretion* ^219^	Increased^220^, ^221^
Norepinephrine	*Increased peripherally* ^222^; reduced action centrally^223^	Increased peripherally; reduced action centrally^224^	*Increased* ^221^
Growth hormone	*Increased* ^61^, ^62^	*Increased* ^225^	Increased^226^
Insulin	*Decreased* ^222^, ^227^	*Increased* ^228^	*Decreased* ^a^ ^221^, ^229^, ^230^, ^231^; impaired secretion
IGF1	*Decreased* ^232^	Decreased^233^	Decreased^234^

Data are compared with healthy (all human studies) or pair‐fed controls. Trends reported from human studies are italicized.

^a^ Compared with non‐cachectic cancer patients.

## Protein malnutrition

Protein malnutrition represents a special case of undernutrition, in which specific adaptations evolved to ensure adequate intake of amino acids essential for growth, reproduction, and survival.^64^ In contrast to simple starvation, which is defined as caloric insufficiency, in protein malnutrition, an imbalance in amino acid content or inadequacy of one or more amino acids drives behavioural and metabolic responses designed to correct the imbalance. Protein malnutrition can refer to a broad range of protein‐deficient diets from total lack of protein content to specific amino acid shortages. For the sake of this review, we will define protein malnutrition as either overall inadequate protein intake—a low protein (LP) diet—or the dietary absence of a single essential amino acid (EAA‐deficient diet), a scenario which best exemplifies the protein‐specific homeostatic circuit. Absence or excess of single amino acids elicit powerful feeding and behavioural effects, which in some cases can exacerbate overall energy imbalance. Imbalances in amino acids are then recovered via the increased catabolism of lean tissues, which may rely upon pathways that increase RMR. This highlights a graded system in which regulation of nutritional composition preferentially drives the response programme over pure caloric content. Below, we discuss the parallel mechanisms that mediate these processes and their interactions with homeostatic systems employed during starvation.

### Ingestive behaviour

Animals fed an LP diet display alterations in appetitive behaviours that vary depending on protein content. For example, rats fed a moderately LP diet, with 8–10% of energy as protein, display sustained hyperphagia, prioritizing normalizing protein levels over caloric homeostasis.^65^, ^66^, ^67^ However, rodents consuming either very LP diet (<8% of energy in rats or <5% in mice) or EAA‐deficient diets become hypophagic, even in the context of negative energy balance.^68^, ^69^ When exposed to such a diet, rats will decrease meal size and increase interfeeding intervals within 20 min of meal onset, mediated by neuronal detection of the EAA imbalance.^70^, ^71^ Within hours, the rats then develop conditioned taste aversion to the deficient diet while also developing preference for the missing amino acid.^72^, ^73^ Thus, despite undernutrition, laboratory rodents will paradoxically sustain hypophagia in the presence of EAA‐deficient foods. When given access to foods containing the missing amino acid, or if the deficient EAA is injected into the brain, feeding behaviour rapidly resumes with preference shown for the nutritionally replete food.^74^ Upon refeeding, rats will even prefer a protein‐free meal to an amino acid‐imbalanced chow, reinforcing the importance of amino acid composition as a driver of food preference.^75^ Chronic hypophagia is therefore driven by aversion to EAA deficiency, not lack of appetite. These observations translate somewhat to human studies, as moderate restriction of dietary protein induces adaptive changes in food intake to restore adequate protein status, but people will not overeat a very LP diet to the point of protein repletion.^76^, ^77^ These findings led to the hypothesis of a ‘protein‐centric’ feeding paradigm, in which dietary amino acid composition is proposed to be the primary determinant of ingestive behaviour, superseding the drive for energy homeostasis.

The sensing of amino acid deficiency during protein malnutrition is complex and involves both the hypothalamus and the anterior piriform cortex (APC), a region involved in olfaction that is amongst the most primitive parts of the mammalian cortex.^78^, ^79^ The APC detection mechanism relies on the accumulation of uncharged transfer RNA, which activates the general amino acid control non‐derepressing kinase 2 (GCN2). Mice and drosophila lacking GCN2 do not detect or avoid EAA‐deficient diets, unless this exposure is prolonged.^78^ GCN2 phosphroylates eukaryotic initiation factor 2 (EIF2A) in APC neurons, initiating a signalling cascade that functions to block general protein synthesis.^78^, ^80^ The net effect of this pathway is to reduce GABAergic inhibition in the APC circuit and increase glutamatergic transmission.^81^ Although it remains unclear which targets of APC mediate the anorectic response, fMRI assessments in rats show rapid activation of both the ventromedial and lateral hypothalamus, two regions involved in feeding behaviours that receive APC axonal projections.^81^, ^82^ However, recent conflicting studies question the GCN2/EIF2A‐dependent mechanism in the APC, suggesting that neuronal sensing of amino acids remain incompletely understood.^83^ A similar mechanism for EAA detection in the MBH was proposed, supported by the blunting of the anorectic response to a leucine‐deficient diet following adenoviral knockdown of GCN2 in the arcuate nucleus of mice.^84^ Although appetite‐regulating neurons in the hypothalamus may directly detect EAA deficiencies, central melanocortin signalling appears to only play a minor role in the acute feeding response. Mice depleted of the MC4R have a slightly attenuated acute anorectic response to EAA‐deficient diet, but neither pharmacologic blockade nor genetic blockade impacts the chronic hypophagia induced by dietary EAA deficiency.^68^ This observation illustrates the mechanistic and behavioural divergence between feeding responses—driven principally by aversion—and appetite, which is preserved.

### Locomotor activity

Rodents fed EAA‐deficient diets develop rapid and sustained anorexia yet display increased foraging behaviours in effort to find foods containing the needed EAA.^81^ This supports the observation that hypophagia observed during EAA‐deficient diet consumption is not reflective of a global decrease in appetitive behaviours. Similar to fasting, both LP diet and EAA deficiency significantly increase locomotor activity compared with rodents fed normal chow.^85^ During the initial EAA‐deficient meal, increased activity corresponds temporally to meal termination and is characterized by digging in their food cup, suggesting that the purpose of this activity is to seek new foods.^86^ Accordingly, this behaviour is rapidly extinguished upon reintroduction of the deficient EAA, in a process dependent upon normal protein synthesis in the APC.^87^

As with the regulation of food intake, the regulation of foraging behaviour because of protein malnutrition is complex. The key site of signal integration appears to be orexin neurons in the lateral hypothalamus, which receive both excitatory projections from the APC and peptidergic projections from the MBH. Disinhibition of APC neurons in response to EAA deficiency then directly activates lateral hypothalamic orexin neurons, which coordinate the locomotor appetitive behaviours (reviewed in Gietzen and Aja ^81^). Karnani and colleagues demonstrate that orexin neurons are also activated by non‐EAAs, which may be increased in the context of EAA deficiency, leading to increased foraging activity.^88^ These authors were further able to show that non‐EAAs at physiologic concentrations were able to overcome glucose inhibition of orexin neurons, providing a mechanistic explanation for the activation of foraging in the EAA‐deficient setting despite adequate calorie intake.^88^ Collectively, it is clear that protein malnutrition induces foraging behaviours similar to that of a starving animal and, combined with a strong preference for amino acid replete food sources, serve to maximize the animal's chances of rectifying nutrient imbalances.

### Resting metabolic rate

The central sensing of amino acid deprivation can regulate energy expenditure and autonomic outflow through mechanisms that are independent of other macronutrients. Dietary leucine deprivation increases thyrotropin‐releasing hormone expression in the hypothalamus, ultimately increasing energy expenditure as indicated by measures of locomotor activity, oxygen consumption, and temperature regulation.^89^ This collective increase in thyrotropin‐releasing hormone observed during leucine deprivation results in SNS activation and autonomic outflow to peripheral tissues that increases RMR and lipid catabolism, as reviewed earlier. Specifically, leucine deprivation increases the expression of the β3‐adrenoceptor, *Adrb3*, as well as *Ucp1* in BAT consistent with sympathetic activation of thermogenesis.^90^, ^91^ Similarly, we found that *Ucp1* expression was increased in mice and rats fed with a valine‐deficient diet, and Guo and colleagues showed that both valine and isoleucine deficiency increase lipid mobilization and energy expenditure suggesting that hypermetabolism is a conserved response to amino acid imbalance.^68^, ^85^ Other investigators demonstrated that leucine deprivation induced the induction of *Ucp1* and other markers of thermogenic activation (generally known as ‘browning’) in WAT, via a CNS pathway and activation of sympathetic outflow.^92^ The evolutionary benefit of this increase in metabolic rate and metabolic reprogramming of adipose tissue in an undernourished animal is not immediately clear, but may be important to balance the energy demands of foraging, via SNS‐mediated activation of lipolysis.^90^

### Fuel utilization and tissue catabolism

When compared with starvation, protein malnutrition is broadly associated with an earlier onset and increased protein and fat catabolism (*Figure* 2B). After being fed a diet deficient in EAA, rodents quickly deplete carbohydrate stores similar to that observed during simple starvation. However, rodents catabolize muscle at a significantly higher rate than their normal chow pair‐fed counterparts, demonstrating that a distinct and independent catabolic pathway is associated with EAA deficiency.^68^ Active muscle catabolism releases EAAs into the blood, thereby providing a source of diet‐limited EAAs and allowing for ongoing protein synthesis to maintain essential physiological processes. Mechanistically, this process is driven by the induction of catabolism‐inducing E3 ubiquitin ligases (MuRF1 and MAFbx) in skeletal muscle. These catabolic proteins are upregulated in the muscle of rodents fed an EAA‐deficient diet after just 3 days and remain elevated after nearly 3 weeks.^68^ To a lesser extent, this increased catabolic state observed in the muscle compartment for EAA‐deficient animals is also observed in fat tissues. Specifically, rodents on an EAA‐deficient diet catabolize fat stores similarly to pair‐fed controls for the first week, but burn fat at a much higher rate after 2 weeks.^68^ This catabolic programme is likely to be influenced both by increased energy expenditure described earlier, along with the actions of circulating glucocorticoids. Although the general trends of hormonal changes associated with protein malnutrition are similar to those found in starvation (*Table* 2), administration of a valine‐deficient diet increased plasma corticosterone compared with pair‐fed control mice and rats.^68^ In response to a leucine‐deficient diet, Xia and colleagues define a novel role of p70 S6 kinase 1 (S6K1) in modulating expression of corticotropin‐releasing hormone in MC4R‐positive hypothalamic neurons. This induction of hypothalamic corticotropin‐releasing hormone expression is essential for stimulating lipolysis in response to leucine deprivation.^93^ Furthermore, glucocorticoids play a well‐established role in mediating skeletal muscle catabolism via the induction of the E3 ubiquitin ligases referenced previously in multiple pathophysiologic conditions.^94^, ^95^, ^96^ Although the dependence of muscle catabolism in response to EAA deficiency upon glucocorticoid elevation has not been directly confirmed, the associative data provide compelling evidence that glucocorticoids serve as a unifying endocrine mediator of macronutrient mobilization. Conversely, the levels of the anabolic hormones insulin and insulin‐like growth factor‐1 are decreased in rodents on an EAA‐deficient diet compared with pair‐fed animals, further shifting the metabolic balance towards catabolism.^68^ In total, EAA‐deficiency results in a sustained catabolic state of peripheral tissues, liberating fat and muscle stores at a faster pace than simple starvation.

## Cachexia

Cachexia is a wasting syndrome associated with a broad range of acute and chronic illnesses, including infection, heart disease, cancer, and chronic inflammatory conditions. Unlike starvation and protein malnutrition, which are dictated by environmental nutrient availability, cachexia results from internal factors and cannot be fully reversed by nutritional supplementation. Cachexia is characterized by the co‐occurrence of anorexia, lethargy, hypermetabolism, and accelerated catabolism.^97^ This programme is the result of inflammatory and metabolic signals that reorient the homeostatic mechanisms employed during starvation and protein malnutrition, establishing a hierarchy of context‐specific afferent signals. That cachexia is conserved across species, and inflammatory conditions suggest that it likely evolved as an adaptive response to life‐threatening illness. Indeed, several studies demonstrate survival benefits of this catabolic state during acute infectious processes.^98^, ^99^ These benefits are traditionally thought to result from the redirection of valuable metabolic resources from the brain and gut to the immune response.^100^ Recent work adds that this metabolic programme aids in tissue tolerance during the immune response, preventing end organ dysfunction.^98^, ^99^ Proinflammatory cytokines are common amongst cachectic conditions and sufficient to drive much of the metabolic physiology. Recent work has expanded the list of afferent mediators contributing to wasting and offered new insights into its aetiology. We contrast the CNS response to cachexia from those of starvation and protein malnutrition and discuss the mediators that drive these divergent programmes.

### Ingestive behaviours

**Figure 3:**
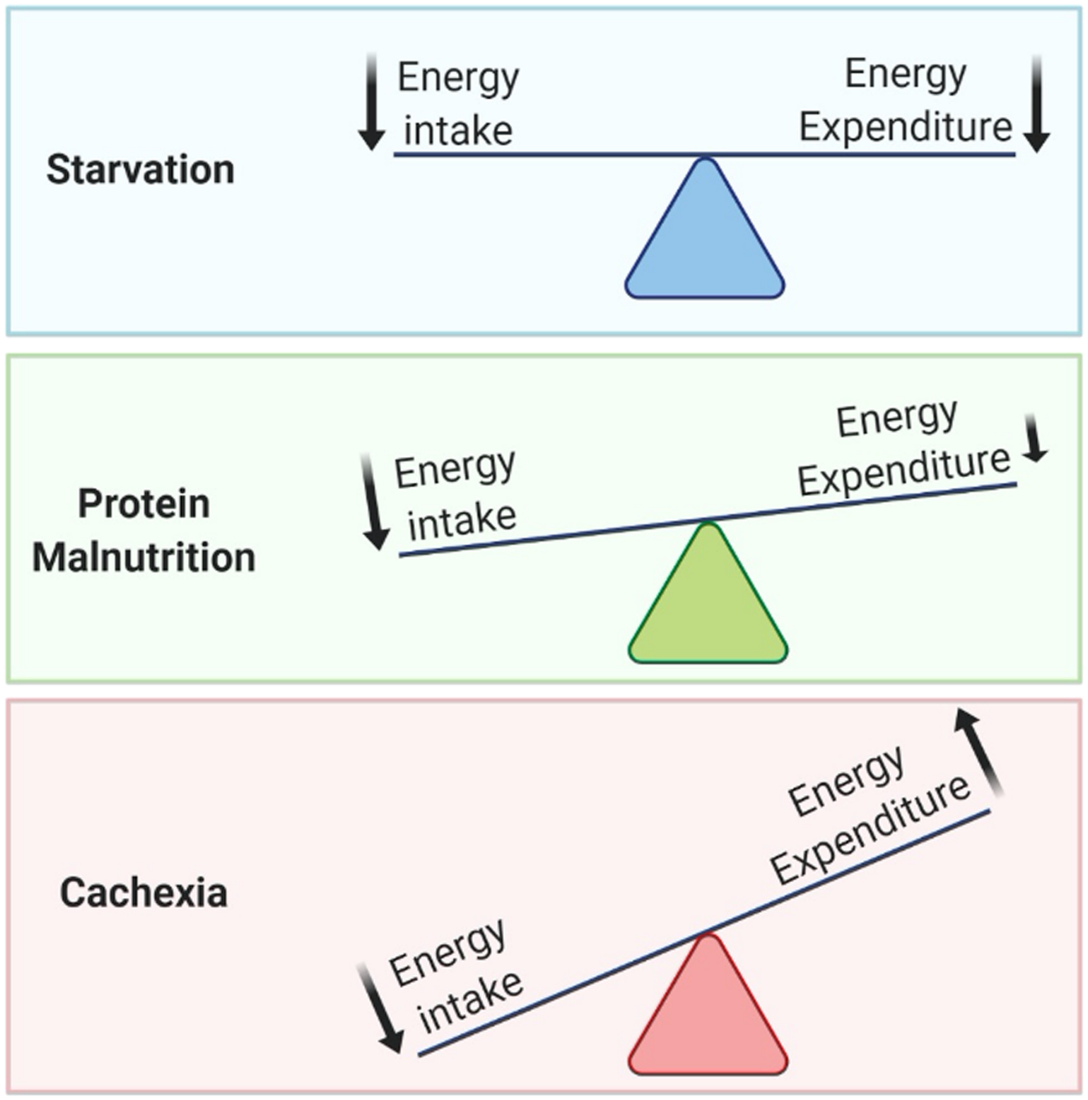
Energy intake and expenditure during starvation, protein malnutrition, and cachexia.

Because of the increase in resting energy expenditure during cachexia, an increase in energy intake would be required to offset the overall catabolic state. However, cachexia induces appetite suppression that amplifies the overall energy deficit (*Figure* 3). In some cases, this presents as frank anorexia, whereas in other cases, a more subtle failure to appropriately increase feeding is observed.^101^, ^102^, ^103^, ^104^ The resistance to the effects of energy debt are most notable after substantial weight is lost, when the hypothalamic feeding rheostat would be under increased pressure to reestablish homeostasis. The disinterest in feeding during cachexia is in direct opposition to what is observed in simple starvation or protein malnutrition, each of which results in behaviours that attempt to restore macronutrient homeostasis.

Multiple systemic and central factors converge to inhibit ingestive behaviours, with the hypothalamus and brainstem thought to be the major sites of signal integration. Principal amongst anorectic factors are inflammatory cytokines, including interleukin‐1β (IL‐1β), tumour necrosis factor (TNF), leukaemia inhibitory factor (LIF), Type 1 interferon (IFN), and prostaglandins.^97^, ^105^ Each of these molecules is independently capable of inducing anorexia when administered either peripherally or directly in the brain. Research in our laboratories and others' over the past two decades demonstrated that a principal site of action for these cytokines is in the MBH, where specialized fenestrated endothelium and localized inflammatory cells allow for the transmission and amplification of peripheral inflammatory signals (reviewed in Burfeind *et al*.^106^). These cytokines then act directly or indirectly to increase signalling through the MC4R, in a fashion similar to leptin.^101^ Pharmacologic or genetic ablation of MC4R signalling reverses cachexia in multiple acute and chronic laboratory rodent models of cachexia.^107^, ^108^, ^109^, ^110^, ^111^, ^112^

More recent work implicates two populations of neurons in the brainstem in the anorectic component of cachexia. The first consists of neurons in the PBN, located in the pons, which relays noxious stimuli from the viscera to the amygdala as a component of the threat circuit. Activation of PBN calcitonin gene‐related peptide (CGRP)‐expressing neurons potently suppresses appetite.^113^ These neurons were activated in two murine models of cancer cachexia and sterile inflammation, and their chemogenetic inhibition was sufficient to reverse the anorexia and weight loss associated with each model.^113^, ^114^ As PBN CGRP neurons are inhibited by hypothalamic AgRP neurons and express MC4R, they may function as a downstream effector of hypothalamic‐mediated cachexia.^115^, ^116^ Given their role in relaying aversive signals, these neurons may also have non‐overlapping influences on cachexia–anorexia, generating redundancy in this system. A second brainstem site involved in the transmission of noxious signals is located in the area postrema, a circumventricular organ most well known for its role in nausea. A population of neurons there expresses the GDNF family receptor alpha like, the only known receptor for growth differentiation factor 15 (GDF‐15), a member of the transforming growth factor beta ligand family that suppresses food intake.^117^, ^118^, ^119^ Elevated serum levels of GDF‐15 are found in multiple cachectic states and serum levels correlate with weight loss in prostate cancer.^99^, ^120^ Treatment of mice with GDF‐15 or implantation with a GDF‐15 overexpressing tumour is sufficient to induce cachexia, whereas treatment with a neutralizing antibody reversed cachexia in multiple murine cancer models.^104^, ^121^, ^122^ Although there is some integration of these anorectic pathways, the existence of multiple disparate mediators represents a level of redundancy found in few biological systems, suggesting that suppression of food intake during illness is an essential response. Indeed, a provocative study by Wang and colleagues demonstrated that improvement in survival in mice with a *Salmonella* infection required fasting‐induced ketogenesis.^98^ Whether decreased nutrient intake provides an adaptive advantage in the contexts of cancer or other conditions remains unclear.

### Locomotor activity

Unlike starvation or protein malnutrition, cachexia is associated with a profound lethargy and decrease in both foraging and non‐foraging locomotor activity. Lethargy is amongst the first signs of sickness, often observed prior to the onset of fever in patients with microbial infections or murine models of sterile inflammation.^123^ From an evolutionary perspective, this response makes sense both to reserve metabolic resources for the fight against infection and to avoid exposure to the elements or predation while in a weakened state. Cachectic rodents neither exhibit the increase in foraging behaviour seen in other states of undernutrition nor develop anticipatory activity when entrained with time‐restricted feeding paradigms.^124^ As described earlier, foraging behaviour is mediated by perifornical/lateral hypothalamic orexin neurons, which receive input from the MBH, APC, and brainstem arousal centres. Orexin neuron activity is decreased in mice treated with lipopolysaccharide and sarcoma‐bearing rats, and intracerebroventricular administration of orexin can restore normal locomotor activity in these models, suggesting that the inhibition of orexin neuron activity underlies sickness‐associated lethargy.^124^ This inhibition appears to be mediated by an increase in the activity of local interneurons that express neurotensin, but the link between inflammation and the activity of these neurons remains poorly described.^124^ Within the MBH, AgRP neuron activity is necessary to engage in foraging behaviour, yet AgRP neuron activity and peptide release are reduced in cachectic rodents.^24^, ^125^, ^126^ As AgRP neurons are known to share reciprocal projections with the lateral hypothalamus and thought to drive foraging via activation of orexin neurons, the loss of AgRP neuron activity may mediate the downregulation of orexin neuron activity. The aforementioned study that evaluated PBN CGRP neurons also found that their chemogenetic inhibition reversed lethargy.^114^ Neurons in the lateral PBN send projections to orexin neurons that then innervate the locus coeruleus, but it remains unclear whether orexin neurons play a role in lateral PBN CGRP‐mediated lethargy or these are two parallel pathways.^127^

Decreased activity in cachectic humans and rodents appears to be multifactorial, involving peripheral mechanisms, as well. Muscles from cachectic mice exhibit decreased mass, strength, and function and exhibit early fatigability.^128^, ^129^ Because inflammatory signalling can drive both skeletal muscle wasting and lethargy, it can be difficult to attribute the decrease in locomotor activity specifically to muscle wasting. However, transgenic mice overexpressing the transcription factor Forkhead box protein O1 (FoxO1), a driver of skeletal muscle catabolism, show reduced muscle mass and spontaneous locomotor activity, suggesting that skeletal muscle loss is sufficient to suppress activity.^130^ Cachexia is further associated with metabolic changes that alter fuel mobilization and utilization. Recent work in mice bearing head and neck cancers shows that decreases in locomotor activity can occur independent of central inflammation and prior to significant muscle loss.^131^ Metabolic phenotyping in these mice revealed significant changes in carbohydrate metabolism associated with lower blood glucose levels and increased skeletal muscle lactate accumulation, positing an additional contribution of metabolic exhaustion to hypoactivity in cachexia.^132^

### Resting metabolic rate

The regulation of RMR in cachexia is most thoroughly studied in the context of cancer, with the majority of studies demonstrating normal or increased energy expenditure in a variety of different cancer types.^133^, ^134^, ^135^, ^136^ Cancer patients frequently have reduced caloric intake and weight loss, so even ‘normal’ energy expenditure should be considered excessive in this context. RMR is related to the degree of cachexia, with elevated energy expenditure documented prior to overt weight loss, sustained through early cachexia, then declining in those most severely affected (refractory cachexia),^133^, ^137^ perhaps because of depletion of available metabolic substrates. Indeed, increased fat oxidation is observed in cancer patients, irrespective of weight loss, and browning of WAT is observed in murine cachexia models and in humans with cachexia.^103^, ^138^ Although direct action of circulating factors (e.g. parathyroid hormone related peptide) can activate thermogenesis and browning of adipose tissue in a subset of cancer types, the most robust and consistent driver of this process is chronic activation of adrenergic sympathetic inputs. A number of studies demonstrate activation of pre‐autonomic neurons in the paraventricular nucleus of the hypothalamus during both the early and late stages of cachexia, suggesting that this is a common mechanism of induction of thermogenesis in this disease.^139^, ^140^ Although few in number, studies that explored the impact of glucocorticoids on human adipose tissue thermogenesis demonstrated an increase in BAT activation by glucocorticoids, suggesting that this is another mechanism driving increased energy utilization during cachexia.^141^, ^142^ Furthermore, the hormonal changes associated with cancer cachexia are characterized by increased release of thyroid hormone as compared with non‐cachectic patients, indicating that this may further increase the RMR^143^ (*Table* 2). Collectively, existing data argue that increased metabolic rate (or lack of compensatory metabolic response to insufficient caloric intake) is an important feature of cachexia, driven by a combination of metabolic inefficiency (‘futile’ metabolic cycles) and tissue reprogramming. However, the afferent signals driving these events, as well as the relative contribution of CNS vs. peripheral mechanisms remain poorly described.

### Fuel utilization and tissue catabolism

As in starvation and protein malnutrition, cachexia is associated with a global shift from carbohydrate to lipid oxidation in both patients and rodent models of disease.^144^, ^145^, ^146^ Levels of both glucose and lipids are frequently elevated in the blood of cachectic patients and rodents, indicating adequate substrate availability, particularly in early stages of cachexia.^147^, ^148^ That hyperglycaemia is common in cancer patients despite the tumour's increased glucose avidity, implies a global metabolic reprogramming favouring lipid as a substrate. The relative fuel utilization can be measured by indirect calorimetry and is expressed as the respiratory exchange ratio (RER)—the ratio between the amount of carbon dioxide generated and the amount of oxygen consumed. Multiple studies show a decrease in RER of cachectic patients and rodents, indicating a preference for lipid oxidation over glucose.^103^, ^144^, ^149^ Evidence from models of early cachexia suggests that this transition occurs before substantial weight loss occurs.^144^ Lipid oxidation appears to be elevated principally in the skeletal muscle, where excess lipid oxidation may be sufficient to drive muscle wasting.^144^, ^150^ However, hepatic lipid oxidation, required for ketone generation, is decreased in multiple models of cachexia, demonstrating a tissue‐dependent reprogramming.^151^, ^152^ This impaired that ketogenesis is hypothesized to be a driver of tissue wasting, but conflicting data exist, with dietary or pharmacologic activation of ketogenesis shown to reverse cachexia in mouse models of lung and pancreatic cancer, but not in a rat sarcoma model or human cancer patients.^151^, ^153^, ^154^, ^155^ The use of proteins as a metabolic fuel is more difficult to measure, as RER does not take them into account, and they generally constitute a small contribution to overall metabolism. Although muscle wasting is a cardinal feature of cachexia, levels of serum amino acids and urinary nitrogen excretion are largely unaltered or paradoxically decreased in cachectic patients and rodents.^156^, ^157^, ^158^ Much of the protein mobilized from muscles during cachexia is thought to provide substrate for the hepatic acute phase response to inflammation, a metabolically costly process involving the synthesis and release of bioactive proteins involved in modifying the metabolic environment of the threatened host. Because the amino acid composition of muscle differs substantially from acute phase reactants, it is hypothesized that this drives further wasting to supply adequate levels of the limiting amino acids.^159^

In comparison with both starvation and protein malnutrition, cachexia is associated with the greatest muscle and fat catabolism relative to the degree of caloric deficiency (*Figure* 2C).^102^, ^160^ Using computational models of cachexia in humans, it is estimated that lipolysis increases by up to 30–80% over baseline,^161^, ^162^, ^163^ while reports suggest muscle catabolism may increase by 40–60%.^164^, ^165^, ^166^, ^167^ Muscle loss in cachexia owes to a combination of reduced protein synthesis and increased protein catabolism.^168^ The relative influence of altered synthesis and degradation to wasting varies amongst studies, with early reports suggesting that decreased synthesis played a dominant role.^169^, ^170^ More recent studies clearly established that cachectic patients retain anabolic potential, with a clinical trial showing net gain in muscle mass with the ghrelin mimetic (anamorelin) in patients with cancer cachexia.^171^, ^172^, ^173^, ^174^, ^175^ Amino acid supplementation in cachectic tumour‐bearing rats increased protein synthesis, yet degradation outpaced gains in protein synthesis.^176^ This catabolic programme in skeletal muscle is mediated through the activation of the ubiquitin proteasome pathway and enhanced *Mafbx*, *Murf1*, and *Foxo1* expression.^177^ Although common to all three states of undernutrition, the ubiquitin proteasome pathway is activated to a greater degree in cachexia than in either starvation or protein malnutrition.^68^, ^102^ The activation of the ubiquitin proteasome pathway in cachexia reflects the influences of direct inflammatory cytokine signalling on muscle, persistently elevated glucocorticoid signalling, and disuse.^178^ Multiple *in vitro* and preclinical studies confirm that inflammatory cytokines, including IL‐1, TNF, and IFNγ, and glucocorticoids, are independently sufficient to induce E3 ubiquitin ligase expression in skeletal muscle, thereby amplifying the catabolic effect of undernutrition.^179^, ^180^, ^181^

Autophagy, the digestion and recycling of cellular contents by the lysosome, also contributes to muscle catabolism. This process is an important component of cellular homeostasis, allowing for the degradation of damaged organelles, toxic protein aggregates, and misfolded proteins.^182^ Autophagy is increased following prolonged fasting in mice, but notably is elevated in the muscles of cachectic human mice as well, as evidenced by increased levels of autophagy mediators BNIP3 and LC3B and the autophagy‐promoting transcription factor FOXO3.^183^, ^184^, ^185^, ^186^, ^187^, ^188^, ^189^ Similar to the ubiquitin‐proteasome pathway, autophagy can be activated in skeletal muscle by metabolic (calorie restriction), hormonal (glucocorticoid), or inflammatory (cytokine) challenges, illustrating the high degree of conservation in muscle‐intrinsic catabolic mechanisms across contexts.^185^ As in skeletal muscle, cardiac wasting can be driven by both the ubiquitin proteasome pathway and increased autophagy. Few extant data support the role of increased MAFbx and MuRF‐1 in hearts from cachectic mice, however, with conflicting reports in mice with cancer cachexia.^102^, ^190^, ^191^ Conversely, autophagy markers LC3‐II, cathepsin L, and beclin are elevated in hearts from rodents with cancer cachexia.^190^, ^192^, ^193^ The relative roles for these pathways in cardiac wasting remain unclear and a topic of active investigation. Ultimately, the cachectic humoral milieu, characterized by increased levels of proinflammatory mediators and glucocorticoids, is able to augment physiologic activation of these catabolic pathways in skeletal and cardiac muscle beyond that of undernutrition alone.

Loss of WAT in cachexia is due to enhanced lipolysis, associated with elevated levels of circulating free fatty acids and glycerol.^194^ Lipolysis is mediated by two enzymes in adipocytes—adipose triglyceride lipase (ATGL), which catalyzes the initial hydrolysis of triglycerides to diacylglycerol, and hormone sensitive lipase, which is responsible for the subsequent hydrolysis of diacylglycerol. Although hormone sensitive lipase is generally considered the main inducible driver of lipolysis, deletion of *Atgl* prevented lipolysis in the B16 melanoma murine model of cancer cachexia.^195^ Lipolysis in cachexia is mediated by increased SNS activation and a host of humoral mediators, including TNF, IL‐6, and zinc alpha glycoprotein, each of which is commonly elevated in the serum of cachectic patients or rodents.^194^ Cachexia is also associated with browning of WAT to promote thermogenesis via the expression of the UCP1.^103^, ^138^ In this way, cachexia modifies adipocyte biology both to induce WAT atrophy via lipolysis and to increase metabolic rate through excess energy dissipation. Fat and muscle catabolism are largely studied as independent events in cachexia; however, several murine studies demonstrate that preventing adipose wasting also reversed skeletal muscle loss.^138^, ^195^ The link between adipose wasting and muscle loss remains unclear but may owe to oxidative stress associated with the increase in fatty oxidation seen in cachectic muscle.^150^ When compared with simple starvation and protein malnutrition, the metabolic programmes of cachexia are broadly more catabolic and energetically inefficient, leading to an increase in resting energy expenditure and depletion of metabolic reserves.

## Conclusion

Throughout our evolutionary history, humans developed behavioural and biochemical strategies to cope with nutrient scarcity in the context of famine. However, starvation was not the only threat to survival associated with undernutrition, as changes in ingestive behaviours and metabolism are seen in the contexts of protein malnutrition and infection or inflammation, as well. Herein, we sought to summarize general macronutrient utilization and tissue catabolism shifts observed amongst these three metabolic states, as well as the associated deviations in neurophysiology and behaviours that serve to rectify (or propagate) nutritional imbalances. In the context of simple starvation and protein malnutrition, the metabolic and neuroendocrine responses induce changes in behaviour and metabolism that facilitate correction of the nutritional deficiencies. Throughout the spectrum of starvation and protein malnutrition, the brain receives both local neuroendocrine signals and distant neuroendocrine signals to interpret the body's overall nutritional state and modulates behaviours and motivations in attempt to balance energy needs, with the requirement to balance amino acid composition eclipsing the drive to maintain overall caloric sufficiency. This hierarchy may seem somewhat surprising and may suggest that EAAs and not simple energy equivalents are the limiting nutritional reagent for organismal survival and replication.

The constellation of metabolic and behavioural responses observed during cachexia represent a highly coordinated series of adaptations designed to survive acute insults by shifting priorities to both combat and tolerate the inflammatory challenge. As systemic infection represented the most salient existential threat to animals in the pre‐antibiotic era, the reorganization of metabolism around disease survival provides a teleological narrative for this paradoxical response to energy depletion. From an evolutionary perspective, humans rarely lived long enough to develop chronic diseases associated with cachexia. Because the metabolic alterations of cachexia, including browning of adipose tissue, skeletal muscle, and adipose catabolism, and elevated basal metabolic rate lead to a severe mismatch in energy balance, it is commonly thought that these responses become maladaptive when engaged over a prolonged period, as during chronic disease. Furthermore, the sickness behaviours of cachexia (including appetite suppression, fatigue, and debility) significantly impact patients' quality of life, stimulating efforts to develop treatments aimed specifically towards reversing cachexia. However, future research may yet show advantages to this physiology in chronic cachectic conditions.

We recognize the important contributions that cognitive, emotional, and hedonic inputs play in feeding motivation during both normal physiology and pathology. Because of the widely varying influences these psychosocial inputs play in both energy metabolism and feeding, we chose to focus solely on the brain's integration of metabolic and neuroendocrine cues during starvation, protein malnutrition, and cachexia, with a particular focus on neurological pathways that are distinct amongst these three metabolic states. Nearly all of the mechanistic data discussed in this review are derived from studies in rodents. It is clear that these responses are likely to be heavily modified by telencephalic inputs in humans, which may provide an additional level of context matching to enhance survival.

In starvation, classical neuroendocrine cues, such as gut‐derived and fat‐derived hormones, are predominantly responsible for organismal metabolic and behavioural outputs. During protein malnutrition, the direct sensing of amino acid imbalances through recently identified neuronal pathways in the APC likely play a direct role in driving peripheral tissue catabolism. The neuroscience of cachexia is defined by the production of cachexia‐promoting factors that are not regulated by nutritional stress alone, but by systemic inflammation because of the underlying disease. The CNS‐based pathways that regulate energy homeostasis during cachexia remains an area of active investigation, but a growing body of evidence demonstrates the capacity of the brain to recognize peripheral mediators of sickness, amplify these signals in circumventricular structures, and modulate energy homeostasis through appetite regulation and neuroendocrine and autonomic control of peripheral tissue metabolism.^97^, ^106^, ^196^ Collectively, the neurophysiology of cachexia exacerbates energy losses, whereas the neurophysiology of simple starvation and protein malnutrition ultimately serve to rectify energy imbalances (*Figure* 3). As evolution is driven by competing pressures, these divergent responses are the result of a natural hierarchy of needs and illustrate the profound impact that disease had in shaping animal physiology. Future investigations into the metabolism and neuroscience of these metabolic states may identify distinct diverging points in our evolutionary history, unique neurobiological pathways of CNS metabolic control, and offer new insights into the physiologic plasticity needed for long‐term survival of a species.

## Funding

This work is supported by the National Cancer Institute grants F30 CA254033 (B.O.), R01 CA217989 (D.L.M.), R01 CA234006 (D.L.M.), and K08 CA245188 (A.J.G.), and the Brenden‐Colson Center for Pancreatic Care (D.L.M. and A.J.G.).
